# Adaptation of Parents Raising a Child with ASD: The Role of Positive Perceptions, Coping, Self-efficacy, and Social Support

**DOI:** 10.1007/s10803-022-05537-8

**Published:** 2022-05-04

**Authors:** Louise Higgins, Arlene Mannion, June L. Chen, Geraldine Leader

**Affiliations:** 1 Irish Centre for Autism and Neurodevelopmental Research (ICAN), School of Psychology, National University of Ireland, Galway, Galway, Ireland; 2 Department of Special Education, Faculty of Education, East China Normal University, Shanghai, China

**Keywords:** Autism spectrum disorder (ASD), Adaptation, Parent, Positive perceptions, Coping, Self-efficacy, Social support

## Abstract

This study explored the adaptation of parents raising a child with an Autism Spectrum Disorder (ASD) specifically the contributory role of positive perceptions, coping, self-efficacy, and social support. One hundred and thirty-six parents of children with a diagnosis of ASD completed a battery of self-report questionnaires via an online survey. Using multiple regression analyses positive perceptions, adaptive coping, self-efficacy, and social support were each a significant contributor to one or more positive adaptation outcomes. Multiple moderated regression analysis found no evidence that these factors were significant moderators between behavioural problems and parental adaptation. The implications of these findings in supporting parents raising a child with ASD are outlined.

## Introduction

Autism spectrum disorder (ASD) is a neurodevelopmental disorder often associated with a broad range of difficulties and co-morbid diagnoses which cumulatively impact on the child’s life and those of their parents and family. Given the increased prevalence of ASD (Fombonne, 2020) and the central role that parents play in a child’s life, it is imperative that there is a clear understanding of what helps parents adapt and manage the challenges associated with their child’s ASD. The current study set out to explore the adaptation of parents raising a child with ASD, specifically the role of positive perceptions, adaptive coping, self-efficacy and social support.

Compared with parents of typically developing children and children with other disabilities the research consistently indicates higher levels of stress and anxiety, and lower quality of life for parents (Barroso et al., 2018; Costa et al., 2017; Foody et al., 2014; Hayes & Watson, 2013; Vasilopoulou & Nisbet, 2016). It is therefore argued that ASD creates unique and complex stressors for families, presenting challenges that are different from other disabilities (Randall & Parker, 1999). Nonetheless, there is now growing evidence that families show resilience and adaptation, and that positive family outcomes are also evident (Bayat, 2007; Bekhet et al., 2012; Halstead et al., 2018; Manning et al., 2011; McStay et al., 2015; Meleady et al., 2020). According to Bayat (2007) many families with a child with ASD demonstrate resilience, with factors such as the characteristics of the child, coping strategies, social support, and the perception of the problem, involved in their adaptation. Research has now begun to identify the factors that exacerbate negative outcomes and those that protect against negative outcomes.

### Double ABCX Model of Adaptation

**Fig. 1:**
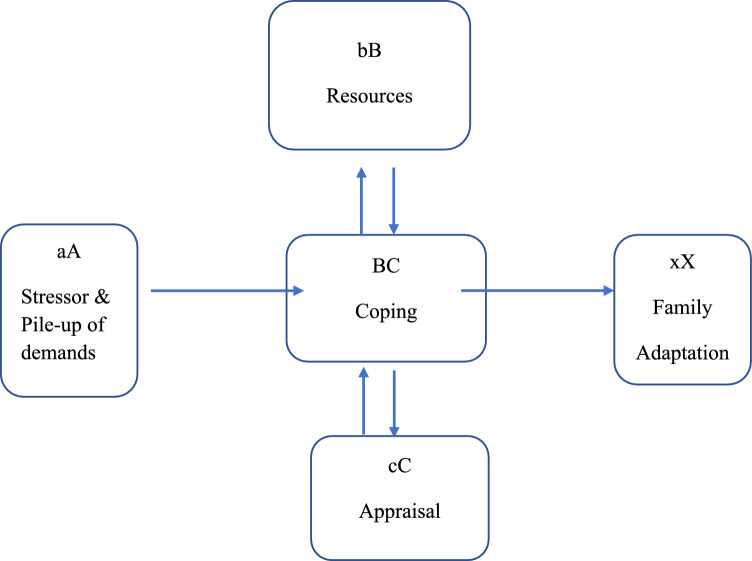
The Double ABCX model based on McCubbin and Patterson (1983)

The Double ABCX model of adaptation (McCubbin & Patterson, 1983) provides a framework for identifying factors which contribute to family outcomes. The application of this model to the adaptation of parents of children with ASD specifies that the adaptation outcome (xX factor) is determined by the interaction between the stressor (i.e., child’s diagnosis) and child characteristics (A factor e.g., behavioural problems), the family’s vulnerability and pile up of demands and stressors (aA factor), family resources (bB factor e.g., social support, parent self-efficacy), perception or definition of the situation (cC factor e.g., positive perceptions), and coping strategies (BC factor e.g., adaptive coping) over time. Adaptation can be viewed on a continuum ranging from positive adaptation to maladaptation. A number of studies have successfully applied the Double ABCX model to help explain the process of raising a child with ASD, with many providing evidence of protective and risk factors in parents’ adaptation (Manning et al., 2011; McStay et al., 2015; Meleady et al., 2020; Miranda et al., 2019; Yu et al., 2018). Figure 1 illustrates this model as used within this study.

### Behavioural Problems

High levels of behavioural problems (e.g., disruptive behaviour; aggressive behaviour; hyperactivity) can be present in children with ASD from an early age (Chandler et al., 2016). The research to date has consistently demonstrated a link between behavioural problems and increased parental stress or depression and anxiety (Barroso et al., 2018; Lindsey & Barry, 2018; Manning et al., 2011; Yorke et al., 2018), lower family quality of life (McStay et al., 2015; Meleady et al., 2020), and lower life satisfaction (Meleady et al., 2020). There is now a strong body of research to indicate that behavioural problems are one of the strongest contributors to parents’ levels of distress (Manning et al., 2011; Yorke et al., 2018).

### Resources

Perceived social support has been identified as a key resource for families with a child with ASD, specifically it has been found to be strongly associated with lower maternal stress (Lindsey & Barry, 2018; Pozo & Sarria, 2014), lower depression & anxiety (Benson & Karloff, 2009; Ekas et al., 2010), greater quality of life (Meleady et al., 2020; Pozo et al., 2014) and higher levels of family functioning (Manning et al., 2011). Halstead et al., (2018) added to this body of research and found that levels of social support had a moderating effect on the relationship between behavioural problems and well-being of mothers who had a child with intellectual and developmental disabilities (IDD), providing strong evidence for social support acting as a protective factor.

There is also emerging evidence that parental self-efficacy (PSE) is a key resource and a positive contributory factor to the outcomes for parents of children with ASD (DesChamps et al., 2020; Garcia-Lopez, et al., 2016a; Rezendes & Scarpa, 2011). Hastings and Brown (2002) in their study on parents raising children with ASD, found that self-efficacy mediated the effect of behavioural problems on mother’s anxiety and depression, whereas there was no evidence of this with fathers. Self-efficacy did however moderate the effect of behaviour problems on father’s anxiety, thus, self-efficacy acted as a protective factor for fathers. Furthermore, Lindsey and Barry (2018) found that parents’ self-efficacy was a moderator in the relationship between internalising behavioural problems and parental distress. They argued that research examining PSE as a moderator is limited and requires further investigation.

### Appraisal

According to Bayat (2007), when parents receive their child’s diagnosis of ASD they initially experience shock and grief, however, a positive change of perspective and new sense of purpose occurs for most parents two years after receiving the diagnosis. Variables that have been researched as forms of appraisal in families of children with ASD include reframing (Manning et al., 2011); benefit finding (Pakenham et al., 2004), positive contributions (Garcia-Lopez et al., 2016a), and positive perceptions (Halstead et al., 2018; Hastings & Taunt, 2002; Sarria & Pozo, 2015). Positive perceptions refer to the parent’s cognitive beliefs about their child with ASD as being a positive contributor to their family. This definition of positive perceptions is focused on the parents’ defining their child with ASD as being a source of fulfilment and happiness, personal growth, and a source of closeness and strength, as derived from a number of key studies (Behr et al., 1992; Hastings & Taunt, 2002). Studies which have examined this concept of positive perceptions have also included different terms to describe a similar concept e.g., positive contributions (Garcia-Lopez et al., 2016a), meaning making, and benefit finding (Pakenham et al., 2004).

Overall, the research to date suggests that positive perceptions about their child may increase positive parent outcomes such as wellbeing and quality of life (Lickenbrock et al., 2011; Meleady et al., 2020; Sarria & Pozo, 2015), protect families from stress and help in their adaptation (Garcia-Lopez et al., 2016a, 2016b; King et al., 2011; Pozo, et al., 2014). Halstead et al. (2018) found that although positive perceptions had a main effect relationship with well-being of mothers, no evidence was found that they functioned as protective factors in moderating the relationship between behaviour problems and wellbeing for mothers raising a child with IDD. Similarly, Meleady et al. (2020) found that while positive perceptions was a unique contributor to family quality of life and life satisfaction, it did not act as a protective factor in moderating the relationship between behaviour problems and parental outcomes. Similar to PSE however, research examining positive perceptions as a moderator is limited and requires further investigation.

### Coping

Studies have demonstrated that problem-focused coping, positive reframing, and coping through spiritual support, are linked with lower stress and increased positive outcomes for parents raising a child with ASD, whereas parents who adopt active avoidance coping strategies and emotion focused coping strategies report more stress and mental health problems (Benson, 2010; Kiami & Goodgold, 2017; Manning et al., 2011; Miranda et al., 2019).

Benson (2014) completed a longitudinal study of mothers raising a child with ASD and found that the use of disengagement and distraction to be related to increased maternal maladjustment over time, and that cognitive reframing reduced the negative effect of problem behaviour on maternal distress. Halstead et al. (2018) found that while practical coping had a positive impact on mothers’ wellbeing regardless of the level of their child behaviour problems, practical coping did not act as a protective factor in moderating the relationship between behaviour problems and wellbeing for mothers raising a child with IDD. The research to date indicates that coping has a complex role with some studies showing it can act as a moderator while others do not. In the context of the current study the term adaptive coping will be used to describe problem-solving coping strategies, including reframing and coping through spiritual support.

### Adaptation

Adaptation is considered to be the outcome of parents’ efforts to achieve a new balance in family functioning. Research has predominantly focused on negative outcomes (e.g., anxiety, depression), with stress as the most widely studied variable in the literature on parental outcomes (Garcia-Lopez et al., 2016a). Nonetheless, there is now emerging research which has included a focus on positive outcomes such as psychological wellbeing, life satisfaction, and family quality of life (e.g., Halstead et al., 2018; Meleady et al., 2020).

### Gaps in Research

There are several limitations to the existing research. Many of the studies fail to distinguish ASD from other disabilities. In addition, most studies explored the main effects of predictor variables on adaptation, whereas it is argued that a multidimensional approach is needed to explore the influence of different factors and the possible role of moderators (Pozo et al., 2014). Furthermore, while it has been found that both positive and negative outcomes co-occur in families raising a child with ASD, only a small number of studies have examined both positive and negative outcomes of adaptation in families. In a similar vein, the research has been limited in examining parental positive perceptions and positive aspects to raising a child with ASD (Sarria & Pozo, 2015). Furthermore, the limited inclusion of fathers has consistently been noted in review studies (Braunstein et al., 2013; McStay et al., 2015; Rankin et al., 2019). Finally, the research is also lacking in the inclusion of parents from lower socioeconomic environments and diverse ethnic and cultural backgrounds (Corcoran et al., 2015; Higgins, 2018; Lickenbrock, et al., 2011).

### Current Study

The study aimed to explore the role of social support, positive perceptions, adaptive coping, and self-efficacy, in the process of parental adaptation, within the context of child behavioural problems, and with reference to the Double ABCX model. Parental adaptation was measured through parental stress, psychological wellbeing, family quality of life, and life satisfaction. The study aimed to address some of the limitations of previous studies by focusing specifically on parents of children of ASD, both mothers and fathers, both positive and negative outcomes, and including those from lower socioeconomic backgrounds. An understanding of the factors that promote parental adaptation is essential given parents’ role in supporting and caring for their child with ASD (Xue et al., 2014). An understanding of potential protective factors can in turn guide policy and practice (Tint & Weiss, 2016).

It was hypothesised that behavioural problems would increase parental stress and decrease family quality of life, psychological wellbeing, and life satisfaction; and that positive perceptions, adaptive coping, self-efficacy, and social support would contribute to parental adaptation in a positive way i.e., decrease parental stress and increase family quality of life, psychological wellbeing, and life satisfaction. Furthermore, it was hypothesised that positive perceptions, adaptive coping, parental self-efficacy, and social support would act as protective factors (i.e., moderators) in the relationship between child behavioural problems and parents’ adaptation. In other words, the association between behavioural problems and parental adaptation will be weaker when parents report higher levels of social support, adaptive coping, parental efficacy, and positive perceptions; in comparison to those reporting low levels of social support, adaptive coping, parental efficacy and positive perceptions.

## Method

### Sample

Participants were included in the study if they were a parent (i.e., biological, adopted, or foster) raising a child aged between 4 and 18 years: who had a primary diagnosis of ASD; two years or longer; and were residing in Ireland. Exclusion criteria were parents of a child with ASD who was residing in residential care. Parents who had more than one child with ASD were asked to answer the questions in relation to their older child within that 4–18 age group.

A total of 232 parents consented to participate in the current study. Ninety-nine parents partially completed the survey. Of these, only parents who were missing data from one or two measures were included in the sample (*n* = 3). The remaining 96 cases were excluded. This left a final sample of 136 parents of children diagnosed with ASD, 116 mothers (85%) and 20 fathers (15%). All were biological parents. Three mothers and fathers were part of the same family, yielding a sample of 133 families with a child with a diagnosis of ASD, all living in Ireland. The data presented in Table 1 indicate the ages, marital status, income, educational level, and employment status of the parents. With regard to the characteristics of the child with ASD (*N* = 133) the age range was 4–18 years with the mean age of 10.5 years, (*SD* 3.8). Of them, 96 were male (73%), 36 were female (27%) and one recorded as non-binary. Parents self-reported on their child’s diagnosis of ASD. The main category of diagnosis was ASD (81%), followed by Asperger’s Syndrome (16%), and PDD_NOS (3%). This diagnosis was reportedly given by public sector multidisciplinary teams (65%); followed by private sector psychologist/ psychiatrist (31%); and other (3%) such as paediatrician. The mean length of time since their child had received their diagnosis was 5 years (*SD* 3.46 years), with a median of 4 years (M = 4; IQR (4)). Twenty percent of parents had more than one child with ASD. Co-morbid diagnosis of intellectual disability (ID) or global developmental delay (GDD) was reported for 32% of the children. In addition, 37% of the children had one or more additional co-morbid diagnosis (e.g., Attention Deficit Hyperactivity Disorder; anxiety). Summary information on the child characteristics are also presented in Table 1.

The sample size was initially determined through a power analysis conducted using the G*Power 3.1.9.4 computer programme. A post-hoc power analyses was also completed to confirm the a priori power analyses using the G*Power 3.1.9.4 computer program (Faul et al., 2007). Given the sample size of 136 participants, a medium effect size (f^2^ = 0.15), with an alpha level of .05 and power of 0.95, was indicated for multiple regression analysis. Furthermore, in relation to the moderation analysis, a post-hoc power calculation was conducted using the danielsoper.com calculator (Soper, 2020). The power of 0.99 was confirmed, indicating a 99.9% chance of finding statistically significant effects.

### Procedure

Prior to launching the survey, the battery of measures was completed by four volunteers to estimate the approximate time for completion and identify any difficulties. They reported that the full completion of measures took between 30 and 40 min, which was in turn specified in the participant information sheet. Participants were recruited through a combination of methods including circulation of a research flyer to autism support groups and organisations nationwide and through a number of national disability services. The flyer contained some information on the topic of the study, the approximate time for completion, along with the survey link. The option of providing assistance with the completion of the survey and/or sending out a hard copy of the questionnaires was also specified on the flyer. Contact information for the researcher was included in the survey along with a list of support organisations. The online survey was launched at the end of November 2019 and closed at the end of February 2020. The survey was administered using LimeSurvey. A number of efforts were made to include the participation of fathers including the researcher and other clinicians encouraging fathers to partake through their clinical work and through contacting autism groups specifically targeting fathers. No renumeration was offered to participants for survey completion.

### Measures

A demographic questionnaire: *‘All about me and my child’* included questions regarding the family’s background including ages, number of children, employment status, education levels, marital status, in addition to information about the child’s diagnosis and co-morbidities, education, and services attending.

#### ASD Diagnosis

The 40-item *Social Communication Questionnaire,* lifetime form (SCQ; Rutter et al., 2003) was used to support the self-reported ASD diagnosis. The SCQ form yields a total score that is interpreted with reference to a cut off score of 15. A number of studies suggest using a cut off score of 10 in order to ensure the inclusion of children who are more higher functioning (Allen et al., 2007; Barnard-Brak et al., 2016). In the current study the range in total scores was 3–33 (*M* = 20.32; *SD* 6.12). One hundred and seven children (79%) scored above the threshold of 15 as originally recommended by SCQ authors, and a further 25 (18%) scored above the adjusted threshold of ten as recommended by Barnard-Brak et al. (2016). This left four (3%) cases that scored below the threshold of ten. No cases were excluded in the analysis. The measure has demonstrated good internal consistency in prior research (Cronbach’s α .85; Meleady et al., 2020) and in the current study (α = .76).

#### Behavioural Problems

The 30-item *Behaviour Problems Inventory-Short form* (BPI-S, Rojahn et al., 2012a) is an informant-based behaviour rating instrument to assess maladaptive behaviours in individuals with intellectual and developmental disabilities, including ASD (Rojahn et al., 2001). The BPI-S measures the frequency (i.e., options include never; monthly; weekly; daily; hourly) and severity (i.e., mild; moderate; severe) of self-injurious behaviours (e.g., self-biting; head hitting) and aggressive/destructive behaviours (e.g., hitting others; destroying things) as well as the frequency of stereotyped behaviours (e.g., rocking, repetitive body movements; twirling/spinning objects). Total scores are obtained for both self-injurious behaviours and aggressive/destructive behaviours; yielding a total frequency score and a total severity score for each. The stereotyped behaviour yields a total frequency score only. Some studies have used the aggressive/destructive behaviour subscale only (e.g., Garcia-Lopez et al., 2016a). In the current study, all subscale totals were calculated however, the frequency totals were used in the regression analyses. In previous studies the internal consistency for the frequency subscales ranged from Cronbach α .70–.89; and for the severity subscales for self-injurious behaviours and aggressive/ destructive behaviours ranged from Cronbach α .68 and .89 respectively (Rojahn et al., 2012b). In the present study the internal consistency for the frequency subscales ranged from Cronbach α .66 (self-injury) and .85 (aggressive/destructive behaviour); and for the severity scores for self-injurious behaviour and aggressive/destructive behaviour were Cronbach α.73 and .86 respectively.

#### Resources (bB)

The 12-item *Multidimensional Scale of Perceived Social Support* (MSPSS; Zimet et al., 1988) was used as a measure of social support. Respondents answer using a 7-point Likert scale ranging from (1) very strongly disagree to (7) very strongly agree. The scale yields a total score and three subscale scores: family; friends; and significant other. Possible scores on MSPSS range from 12 to 84, with higher scores indicating greater perceived support. Internal consistency has been good in prior studies (α = .91; Alon, 2019) and in the current study (α = .92). For the main analysis the total score was used.

The 17-item *Parenting Sense of Competence* (PSOC; Gibaud-Wallston & Wandersman, 1978; Johnston & Mash, 1989) assessed parents’ sense of their own competence using two broad scales: efficacy and satisfaction. Items are answered using a six-point Likert scale ranging from (1) strongly disagree (6) to strongly agree. The PSOC total score is the sum of efficacy and satisfaction subscale scores. Scores can range from 17 to 102. Higher scores indicate greater parenting sense of competence. This scale has been used in some related studies (e.g., Garcia-Lopez et al., 2016a; Lindsey & Barry, 2018). The internal consistency has been good in prior studies (α = .78; Garcia-Lopez et al., 2016a) and in the current study (α = .82). For the main analysis the total score was used.

#### Appraisal of the Stressor (cC)

The 50-item *Positive Contributions Scale* (PCS) from the Kansas Inventory of Parental Perceptions (KIPP; Behr et al., 1992) was used to measure parental perceptions of the positive contributions their child with ASD has made to them and their family. Items in this measure are rated on a four-point Likert scale ranging from (1) strongly disagree (4) to strongly agree. The PCS total score ranges from 50 to 200. Higher scores are associated with parents’ stronger positive perceptions of their child. The PCS has been used in a number of related studies (e.g., Garcia-Lopez et al., 2016a; Kayfitz et al., 2010). The internal consistency has been good in prior studies (α = .95; Hastings et al., 2005a) and in the current study (α = .95). For the main analysis, the total score was used.

#### Coping (BC)

The 30-item *Family Crisis Oriented Personal Evaluations Scales* (F-COPES), (McCubbin et al., 1991) was used to assess coping strategies employed by the family when faced with problems or difficulties. Respondents rate how well the statements describe their own family’s response to difficulties on a scale from (1) strongly disagree to (5) strongly agree. A total coping score ranges from 30 to 150. Higher scores indicate better problem-solving and behavioural responses. The FCOPES has been used in a number of related studies (e.g., Greer et al., 2006; Hastings et al., 2002) with good internal consistency reported (α = .77–.86; Hastings et al., 2002). In the present study the internal consistency for the total score was good (α = .87).

#### Parental Adaptation (xX)

The 18-item *Brief Psychological Wellbeing* (BPWB; Ryff, 1989; Ryff & Keyes, 1995) was used as a measure of parental wellbeing. Eighteen items are grouped into six subscales of autonomy; environmental mastery; personal growth; positive relations with others, purpose in life, and self-acceptance. Items are answered using a seven-point Likert scale ranging from (1) strongly agree (7) to strongly disagree. Scores range from 18 to 126 with higher scores indicating higher levels of psychological wellbeing. The scale has been used in related studies (e.g., Garcia-Lopez et al., 2016a, 2016b; Pozo et al., 2014). The internal consistency of the total score is good (α = .84; Ryff & Keyes, 1995) and in the current study (α = .85). For the main analysis the total score was used. Two participants did not complete this scale.

The 25-item *Family Quality of Life Scale* (FQOLS) also known as the Beach Center Family Quality of Life Scale (Hoffman et al., 2006) was used to measure parents’ perceptions of their satisfaction with different aspects of family quality of life. It consists of five subscales: family interaction; emotional well-being; parenting; physical/material well-being; and disability-related support. The responses are measured using a five-point Likert scale ranging from (1) very dissatisfied to (5) very satisfied. The total scores range from 25 to 125 with higher scores indicating greater satisfaction with quality of life. The measure has been used in related studies (e.g., Pozo et al., 2014; Staunton et al., 2020). Internal consistency for the total score is good (α = .88; Hoffman et al., 2006) and for the current study (α = .96). For the main analysis the total score was used.

The 5-item *Satisfaction with Life Scale* (SWLS; Diener et al., 1985; Pavot & Diener, 2008) was used as a measure of parents’ satisfaction with their life as a whole. Items are answered using a seven-point Likert scale ranging from (1) strongly disagree to (7) strongly agree, with a higher score indicative of greater life satisfaction. The scale has been used in related studies (e.g., Cohen et al., 2015; Halstead et al., 2018) with good internal consistency reported (α = .87; Halstead et al., 2018) and in the current study (α = .91). Three participants did not complete this measure.

The 36-item *Parenting Stress Index-Short Form* (PSI-SF; Abidin, 1995) was used to measure overall levels of parenting stress. Items are answered using a 5-point Likert scale ranging from “strongly agree” to “strongly disagree”, with higher scores indicating a higher amount of stress. A total score (i.e., overall level of parenting stress) was calculated in addition to the parental distress subscale (PD) which measures the distress experienced by parents due to personal factors, or life restrictions due to the demands of childrearing. The internal consistency has been reported as good in related studies (α = .87 for PD scale; Garcia-Lopez et al., 2016a). The internal consistency was good for the current study for both the total score and the PD subscale (α = .92).

## Results

### Data Analysis

Statistical analysis of the data collected was conducted using SPSS IBM (version 26). All data were examined descriptively, and preliminary analysis completed to examine the distribution of variables. Correlations were used to identify the relationship between the variables examined. One-way ANOVAs, t-test, and correlations were carried out to examine the relationships between demographic variables and parental adaptation in order to identify potential covariates for the regression analysis. Hierarchical multiple regression analysis was carried out to test the hypothesis that positive perceptions, adaptive coping, parental self-efficacy, and social support were contributing significantly to parental adaptation while controlling for co-variates and behavioural problems. Furthermore, moderated multiple regression analysis was completed to test the hypotheses that these factors acted as protective factors moderating the relationship between behavioural problems and each adaptation outcome. The moderation analyses were completed using the PROCESS programme of mediation, moderation, and conditional analysis (Hayes, 2018).

### Preliminary Analysis

Initially the data was managed by completing tests of normality and screening the data for any missing data, irregularities, and significant outliers. The distribution of variables was examined by using the Kolmogorov–Smirnov Test. Those that yielded a significant result were examined and significant outliers removed so that they yielded a non-significant result and the histograms yielded reasonable normal distributions. This resulted in some small variations in the sample size for certain variables. The one exception was the BPI-S measure. The results were positively skewed with most parents recording relatively few behavioural problems. Data on atypical behaviour such as that gathered with the BPI are often positively skewed (Meleady et al., 2020; Rojahn et al., 2012a, 2012b).

With reference to the adaptation measures over half of parents (52%) reported overall levels of parental stress in the clinically significant range (i.e., over the 90th percentile). Using qualitative descriptors for family quality of life, the majority of parents are indicating a good- high quality of life rating whereas 4% reported a low/poor quality of life. The life satisfaction mean score indicates an average rating of life satisfaction in accordance with the scoring criteria by Pavot and Diener (2013).

Parental stress was positively associated with the age of the child with ASD (*r* = .19, *p* < .05); and number of children with ASD (*r* = .17, *p* < .05). Parental stress and distress levels (as measured by the PSI-SF i.e., total score and score for personal distress subscale) were significantly higher for families of lower income (i.e., below €30,000) than those of higher income (i.e., €60–89,000). This was statistically significant at the *p* < .05 level (*F* (3, 131) = 3.52, *p* = .017), (*F* (3, 131) = 3.19, *p* = .026) with a medium effect size. Family quality of life (FQOl) was significantly associated with marital status, in that FQOL was significantly lower for single parents (*M* = 69.23; *SD* 19.60; *n* = 13) than those who were married (*M* = 87.60; *SD* 17.66; *n* = 91). This was a significant difference at the *p* < .05 level (*F* (4, 129) = 3.12, *p* = .017), with a medium effect size. Similar significant results were found for psychological well-being and marital status (*F* (4, 129) = 3.23, *p* = .015), and parental distress and marital status (*F* (4, 130) = 2.64, *p* = .037). Single parents reported significantly lower psychological wellbeing and higher levels of parental distress than parents who were married. These demographics were included in the main regression analyses as covariates for specific models.

### Testing of Hypotheses

Pearson correlations were used to explore the associations between parental adaptation and the variables included in the study. In Table 2 the results from the correlational analysis between behavioural problems with each adaptation measure are illustrated. As hypothesised behavioural problems were consistently associated with increased stress levels. The strength of the relationships varied from medium to large. Furthermore, as hypothesised behavioural problems had a negative association with positive adaptation. Specifically, aggressive/destructive behaviour was associated with a reduction in life satisfaction (*r* =  − .210; .200; *p* < .05); self-injurious behaviour associated with a reduction in FQOL (*r* =  − .307; − .319; *p* < .01), and stereotyped behaviour associated with lower levels of psychological wellbeing (*r* =  − .171; *p* < .05). Parental stress was associated with all other adaptation measures in that the higher the stress levels the lower FQOL, psychological wellbeing and life satisfaction.

Table 2 also indicates the results of correlational analysis conducted between the variables of specific interest and parental adaptation. As hypothesised, positive perceptions, adaptive coping, self-efficacy and social support each had a significant positive association (i.e., medium to large) with FQOL, life satisfaction, and psychological well-being. Whereas positive perceptions, adaptive coping, self-efficacy and social support each had a significant negative association with parental stress. The strength of the correlations ranged from medium to large (*r* = .32 to *r* = . 68). in accordance with criteria by Cohen (1988).

### Regression Analysis

Further to the correlation matrix, the variables which contributed to parental adaptation were explored through hierarchical multiple regression analysis for each adaptation measure. This analysis examined if positive perceptions, adaptive coping, social support, and parental self-efficacy contributed to parental adaptation in a positive way while controlling for certain demographic variables and behavioural problems. Relevant demographic variables were selected for inclusion where they had been found to have a significant association with specific parental outcomes. The adaptation measure was entered as the criterion variable. The predictor variables were then entered in the following order: any demographic variable found to be associated with the specific adaptation measure were entered in the first block, second behavioural problems were added (i.e., behaviour frequency scores), and finally positive perceptions, social support, adaptive coping, and self-efficacy were added in the third block. Multicollinearity was not present in the data. Pearson’s correlation statistics for predictor variables were less than .7 (Table 2). The variance inflation factor (VIF) scores were less than 10 (ranged from 1.0 to 2.4) and tolerance scores were greater than .1 (ranged from .42 to .98).

The first model on parental stress (Table 3) controlled for family income, age of child, more than one child with ASD, and behavioural problems, and explained 65% of the variance in parental stress (*F* (10, 119) = 24.89, *p* < .001, *R*^2^ = .68, adjusted *R*^2^ = .65). Step three of this model which included positive perceptions, adaptive coping, self-efficacy, and social support, significantly contributed to the model, uniquely explaining 33% of the variance in parental stress (Δ*R*^2^ = .33, Δ*F* change (4,119) = 30.12, *p* < .001). In this model, the following five factors were statistically significant and in order of strength: parental self-efficacy (*β* =  − .33, *p* < .001), aggressive/destructive behaviour (*β* = .30, *p* < .001), social support (*β* =  − .24, *p* < .01), more than one child with ASD (*β* = .19, *p* < .001), and family income (*β* =  − .16, *p* < .01).

The second model on FQOL (Table 4) controlled for marital status and behavioural problems and explained 57% of the variance in FQOL (*F* (8, 121) = 22.37, *p* < .001, *R*^2^ = .60. adjusted *R*^*2*^ = .57). Step three of this model which included positive perceptions, adaptive coping, self-efficacy, and social support, significantly contributed to the model, uniquely explaining 50% of the variance in FQOL (Δ*R*^*2*^ = .50, Δ*F* change (4,121) = 37.42, *p* < .001). In this model, the following five factors were statistically significant and in order of strength: social support (*β* = .38, *p* < .001); parental self-efficacy (*β* = .28, *p* < .001), adaptive coping (*β* = .23, *p* < .05), self-injurious behaviour (*β* = − .18, *p* < .05), and aggressive/destructive behaviour (*β* = .15, *p* < .05).

The third model on psychological wellbeing (Table 5) controlled for family income and behavioural problems and explained 53% of the variance in psychological wellbeing (*F* (8, 121) = 19.05, *p* < .001, *R*^2^ = .56. adjusted *R*^2^ = .53). Step three of this model which included positive perceptions, adaptive coping, self-efficacy, and social support, significantly contributed to the model, uniquely explaining 52% of the variance in psychological wellbeing (Δ*R*^2^ = .52, Δ*F* change (4,121) = 35.47, *p* < .001). In this model, the following four variables were statistically significant and in order of strength: adaptive coping (*β* = .41, *p* < .001), parental self-efficacy (*β* = .41, *p* < .001), stereotyped behaviour (*β* =  − .16, *p* < .05), and positive perceptions (*β* =  − .16, *p* < .05).

The fourth model on life satisfaction (Table 6) controlled for behavioural problems and explained 36% of the variance in life satisfaction (*F* (7, 108) = 10.26, *p* < .001, *R*^2^ = .40, adjusted *R*^*2*^ = .36). Step two of the model which included positive perceptions, adaptive coping, self-efficacy, and social support, significantly contributed to the model, uniquely explaining 35% of the variance in life satisfaction (Δ*R*^2^ = .35, Δ*F* change (4,121) = 15.53, *p* < .001). In this model the following two variables were statistically significant and in order of strength: positive perceptions (*β* = .28, *p* < .01) and social support (*β* = .22, *p* < .05).

### Moderation Analysis

**Fig. 2:**
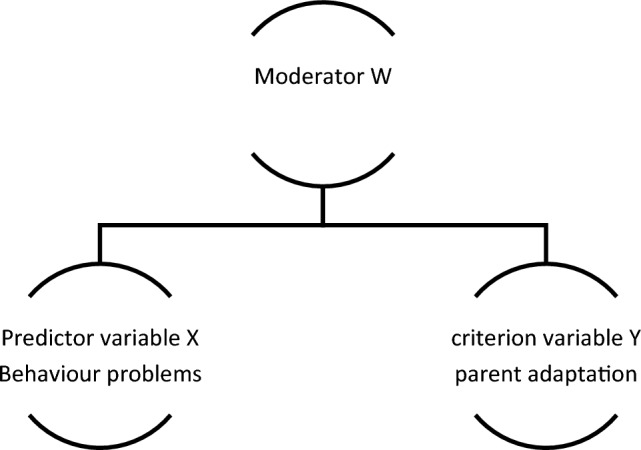
Moderation analysis model

In order to assess the hypotheses that positive perceptions, adaptive coping, parent self-efficacy and social support moderate the relationship between behavioural problems and parental adaptation, a moderated multiple regression was performed using the PROCESS procedure for SPSS version 3.4.1 (Hayes, 2018). The key predictor in each analysis was behavioural problems (i.e., behavioural frequency) entered as the X variable. Parental adaptation was entered as the Y variable. This was carried out four times for each adaptation outcome. The moderator was entered as the W variable. This was carried out four times for each proposed moderator (i.e., coping, social support, positive perceptions, and parental self-efficacy). Covariates were added also where applicable. In the moderation analysis, the bootstrap procedure with 5000 repetitions was used to verify the aforementioned variables, with a confidence interval of 95%. Figure 2 demonstrates the role of the moderator.

The moderators were entered individually both as a main effect and as an interaction variable with behavioural problems. The results from each of those analysis indicated that none of the four variables of interest functioned as a moderator variable in the relationship between behavioural problems and parent adaptation outcome (See Tables 7, 8, 9 and 10). These hypotheses were not supported. Positive perceptions, adaptive coping, parental self-efficacy, and social support were not significant moderators of the effect of behavioural problems on any of the parental adaptation measures.

## Discussion

### Contributors to Parental Adaptation

The current study set out to explore the adaptation of parents raising a child with ASD, specifically the role of positive perceptions, adaptive coping, self-efficacy and social support, with reference to the Double ABCX model. The results indicate that over half (52%) of parents are experiencing clinical levels of stress in relation to their parenting. This finding is well founded in the literature and supports the finding that raising a child with ASD has inherent stressors (Foody et al., 2014; Giovagnoli et al., 2015; Kiami & Goodgold, 2017). Nonetheless alongside these levels of parental stress, the majority of parents are indicating a good-to-high FQOL, with just 4% reporting a low or poor FQOL. Furthermore, the mean life satisfaction score for parents indicates an average rating of life satisfaction. The analyses highlight that parental adaptation as measured in this study yielded co-existing positive and negative adaptation outcomes. This is significant given that the preponderance of research to date has focused more exclusively on negative outcomes.

Many of the hypotheses were supported. Behavioural problems regardless of type, severity or frequency, were all consistently associated with increased levels of parental stress. This finding is consistent with an extensive body of research which has consistently found a strong association between behavioural problems and parental stress (Barroso et al., 2018; Yorke et al., 2018). In addition, specific categories of behaviour problems showed significant negative associations with certain adaptation outcomes (e.g., aggressive/destructive behaviour with life satisfaction, self-injurious behaviour with FQOl, and stereotyped behaviour with psychological wellbeing). These findings were all in the expected direction given the previous research findings that has found behavioural problems to impact negatively on psychological wellbeing and quality of life (Blacher & McIntyre, 2006; Sarria & Pozo, 2015; Vasilopoulou & Nisbet, 2016).

As hypothesised positive perceptions, adaptive coping, self-efficacy and social support each had a positive association with FQOL, life satisfaction, and psychological well-being. Whereas each of these factors had a significant negative relationship with parental stress. These findings are in all in line with the literature to date (Kayfitz et al., 2010; Pozo et al., 2014; Sarria & Pozo, 2015). Furthermore, as hypothesised the factors of positive perceptions, adaptive coping, parental self-efficacy (PSE), and social support, were found to be significant contributors to one or more adaptation outcome even while controlling for specific demographics and behavioural problems.

With regard to parental stress both PSE and social support were significant contributors. These findings add to the body of research that has found social support to be a contributor to reduced stress levels (Lindsey & Barry, 2018; Pozo & Sarria, 2014). Thus, parents who perceive they have adequate social support are more likely to report reduced parental stress. While research has been more limited on PSE, this now provides further evidence that PSE contributes to parent outcomes positively. Furthermore, the study adds to the literature by identifying PSE as the strongest and most significant contributor to lower parental stress. Aggressive/destructive behaviour was also found to be a significant contributor to parental stress which is an expected finding given the literature, as this aspect of behaviour can challenge and cause most stress for parents (Blacher & McIntyre, 2006; Lecavalier et al., 2006; Manning et al., 2011; Tomanik et al., 2004). While not hypothesised having more than one child with ASD and lower family income were also found to be significant contributors to parental stress. This is in line with studies that have found the increased demands for those with multiple children with ASD and the increased costs associated with raising a child with ASD (Dillenburger et al., 2010; Roddy & O’Neill, 2020).

With regard to FQOL, social support, PSE, and adaptive coping were each found to be significant positive contributors. This finding further adds to the evidence of the importance of social support (Pozo et al., 2014), PSE, and adaptive coping (Pozo et al., 2014) as contributors to positive parental outcomes in this case FQOL, where to date research has been limited on this as an adaptation outcome. Both self-injurious behaviour and aggressive/destructive behaviour were also found to be significant contributors to FQOL which was in line with existing research findings regarding behavioural problems (McStay et al., 2015; Meleady et al., 2020; Pozo et al., 2014).

With regard to psychological wellbeing, adaptive coping and PSE were found to be significant positive contributors. The identification of adaptive coping as a positive contributor is consistent with previous studies (e.g., Benson, 2012, 2014; Halstead et al., 2018). While PSE has been found to be a positive contributor to mental health outcomes such as anxiety and depression (Hastings & Brown, 2002), it is perhaps not surprising that it was found to be a positive contributor to psychological well-being. Other significant contributors but to a lesser extent include stereotyped behaviour, and positive perceptions. In line with previous research behavioural problems have been shown to be a negative contributor to parental outcomes such as psychological wellbeing and mental health (Blacher & McIntyre, 2006; Manning et al., 2011).

While in previous studies positive perceptions was found to be a positive contributor to psychological wellbeing (Sarria & Pozo, 2015), in the current study it was found to be a negative contributor. This is an unexpected finding given the research to date and given the earlier analysis found a significant and positive association between positive perceptions and psychological wellbeing. Possible explanations could be attributable to the model of analysis, and due to the interaction of multiple variables which can occur in hierarchical multiple regression analyses. It is also possible that there may be in fact this negative contribution which may be due to the possibility that seeing your child’s positive contributions may result in a parent being more aware of their difficulties and disabilities which in turn may lead to reduced wellbeing.

With regard to life satisfaction, positive perceptions and social support were found to be significant contributors. While this finding is not surprising given that previous studies have found social support to be a significant contributor to positive parental outcomes such as life satisfaction (Halstead et al., 2018; Meleady et al., 2020), this is of significance given that research which has included life satisfaction as a parental outcome and positive perceptions as a contributory factor is still in its infancy.

Thus, in summary, the analysis of parental adaptation supports these four factors as being important and significant contributors to positive parental outcomes. PSE was demonstrated to be a positive contributor to parental stress, FQOL and psychological wellbeing and moreover was the strongest predictor for parental stress and psychological wellbeing. Social support was also a significant contributor to parental stress, FQOL, and life satisfaction and the strongest predictor to FQOL. Adaptive coping was a significant contributor to FQOL, and psychological wellbeing. Finally, positive perceptions was the strongest contributor to life satisfaction. The findings are overall consistent with previous research (e.g. Manning et al., 2011; Sarria & Pozo, 2015), however the current study has added to the body of research while also highlighting new findings including variables that have been less studied (i.e., PSE, positive perceptions, FQOl, and life satisfaction). The analysis found that behavioural problems contributed to certain parental adaptation outcomes, however of interest is that certain categories of behavioural problem contributed to specific adaptation outcomes. This is a significant finding which suggests the potential importance of examining different categories of behaviours. None of the behavioural problems was found to contribute to life satisfaction. While it was anticipated that behavioural problems would have contributed higher and more significantly to adaptation outcomes overall, it is possible that the behavioural measure may have been too narrow in focus and not broad enough to include all possible behaviour problems that occur in this population.

### Evidence of Factors as Moderators

The hypotheses that positive perceptions, adaptive coping, PSE, and social support would each act as a moderator in the relationship between behavioural problems and parent adaptation was not supported. The research has been limited in the exploration of such protective factors and the evidence mixed to date. However, the results are similar to Meleady et al. (2020) who found that positive perceptions and social support did not act as moderators. Whereas Halstead et al. (2018) found social support to have a moderating effect on the relationship between behaviour problems and parental stress and parental wellbeing. While the research is limited with PSE, it has been found to be a moderator between behavioural problems and anxiety in fathers (Hastings & Brown, 2002).

While the results did not support these hypotheses there are two possible explanations. It is possible that the behavioural measure may have been too narrow in focus and may not have captured the full range of behavioural problems that can occur in this population. The results from the behavioural measure were low with the majority of parents reporting low levels of behavioural problems. Furthermore, it is clear from the previous analysis that these factors are contributing to parental adaptation, just not in the role of a moderator. So, while the current study did not identify a protective role (i.e., impacting when there are high levels of behaviour problems), it is possible that they are contributing to parental adaptation regardless of the level of behavioural problems.

The results altogether highlight the usefulness of the double ABCX model in understanding the adaptation processes and the interplay of factors when facing the potential challenges such as those in raising a child with ASD. It allowed for the investigation of positive and negative outcomes and enabled the examination of variables which contributes to the understanding of the resources that influence adaptation positively. This is consistent with previous research that has applied this model to examining adaptation in families of a child with ASD (e.g., Manning et al., 2011; Meleady et al., 2020; Pozo et al., 2014).

### Implications

There are several implications for these findings both in terms of clinical service provision and policy development. Given the high levels of parenting stress it is imperative that clinical services address this as part of their core work, through supports and direct intervention such as mindfulness-based stress reduction (Bazzano et al., 2015; Cachia et al., 2016). This is important as high stress levels can exacerbate behavioural problems and ASD symptom severity (Rodriguez et al., 2019; Yorke et al., 2018), reduce the effectiveness of interventions and result in poorer outcomes (e.g., Osborne et al., 2008; Reyno & McGrath, 2006). In addition, given the strong association of behavioural problems with parental stress and other adaptation outcomes, addressing behavioural problems should be another core component of clinical services for families with a child with ASD. Providing skills training for parents to address these difficulties and at an earlier stage is likely to be beneficial. Furthermore, this could serve to indirectly reduce parental stress while also supporting and building PSE and positive coping skills.

The findings also support the use of a strength-based approach in working with families of a child with ASD. Conveying the message that families have the potential to positively adapt may be helpful for parents especially those new to the diagnosis. Murphy and Tierney (2014) found that parents new to the diagnosis wanted to hear positives in addition to the challenges. Furthermore, the Double ABCX model provides a useful framework for assessing adaptation, family stressors, resources, and areas of support need. In addition, using measures that capture family strengths, positive experiences and outcomes (e.g., FQOL, PCS) could be used in addition to using measures which are problem focused.

Given the positive contribution of PSE, adaptive coping, social support, and positive perceptions to parental adaptation, they each represent possible targets in clinical service provision. These are factors that can be promoted, facilitated, and encouraged in a number of ways. Increasing PSE and ensuring that parents feel confident in their role parenting their child with ASD is likely to be achieved directly and indirectly through skills training interventions and other supports (e.g., social support). Social support should be promoted to all parents through several avenues such as facilitating access to local support groups, providing psychoeducational groups within service, linking with national organisations; signposting to online supports, and referring those who may need to access further supports such as family support services and/or respite. As social support was measured through perceived social support it is also possible that helping parents perceive the environment as helpful will equally benefit parents’ adaptation.

Facilitating a positive focus can occur through services adopting a perspective oriented towards the child’s achievements and other positive aspects of his/her development, and family functioning, in addition to the challenges and problems. In a similar vein, Manning et al. (2011) suggest the possibility of facilitating positive perceptions through exposure to other family’s positive outlook about their child with ASD. Singer et al. (1999) found that several parents reported that contact with a positive attitude in other families who have a child with a disability helped them to alter their own attitude in a positive direction. In addition, clinicians working with parents need to be aware of the coping strategies that parents use to manage the demands of parenting and in turn support them in their use of adaptive coping strategies (e.g., acquiring help and support, reframing, seeking spiritual support) over avoidant coping strategies.

The cost of raising a child with ASD is significantly greater than for those with a typically developing child (Dillenburger et al., 2010), and thus it is not surprising that families raising a child with ASD can experience a financial burden specific to the care needs of their child, which is further exacerbated for those families with multiple children with ASD (Roddy & O’Neill, 2020). The current study adds to this by highlighting those within the population at further risk in terms of increased stress levels due to certain demographics (i.e., those from low income families, and those with multiple children with ASD). These groups are therefore likely to need an elevated level of support.

### Limitations

There were several limitations to this study that need to be taken into account when interpreting the results. First as this was a cross sectional study it prevented any conclusions regarding the directionality of relationships identified. The limited number of fathers participating in the study was a further limitation, especially as prior research which has managed to include both has found that mothers and fathers show different patterns of stress (Foody et al., 2014), different use of resources, and differences in their adaptation to parenting a child with ASD (Hastings et al., 2005a, b; Pozo et al., 2014).

Another factor which needs to be considered is the representativeness of the sample of parents raising a child with ASD. The majority of parents were Irish mothers who were in a relationship. Furthermore, given the study used a convenience sample, the sample may overrepresent engaged and motivated parents who may have differed from those who chose not to participate. The length of the survey and time taken for completion (i.e., 30–40 min) may have proved challenging to parents. This may have been the case for the ninety six parents who only partially completed the survey.

Another limitation is in relation to some of the measures used and the self-report nature of these measures. The behavioural measure used yielded low levels with many parents reporting low levels of behaviours. This measure may be more specific to those with ASD with co-morbid ID which in this current study was just 32% of the overall sample. The measure may not have detected the broader range of behavioural problems especially in those higher functioning children. This study relied on self-report measures from the same single source rather than any independent assessments or from other sources. In addition, the study did not examine the impact of other life stresses, or what is referred to in the Double ABCX model as pile up demands. It is possible that many of these families may have been experiencing other stressors in their life which were not captured, and which may have been impacting on their adaptation. Finally, it could also be considered a limitation that there was a lack of confirmation of the ASD diagnosis by a professional as part of the procedure.

Future research would benefit from longitudinal studies with more diverse samples including fathers, using a broader behaviour measure, and multi-informant measures particularly on critical variables such as child behavioural problems and FQOL. Investigating the possibility of social support, adaptive coping, positive perceptions, and self-efficacy, functioning as mediators in the role between behavioural problems and parental adaptation should be explored. In addition, research should also examine the role of other child characteristics (e.g., their strengths) in parents’ adaptation. Given the difficulties in including fathers, future research could consider using other recruitment techniques such as snowballing. Other types of methodologies may also prove to be more fruitful such as qualitative methods e.g., focus groups.

## Conclusion

The findings support the well-established association between parenting stress and behaviour problems and the high levels of parental stress amongst this group. Despite this, there is evidence that there are co-existing indicators of positive adaptation in the form of family quality of life and life satisfaction. The role of positive perceptions, adaptive coping, self-efficacy, and social support in parents’ adaptation was investigated and evidence found that these factors contributed positively to one or more adaptation outcomes, however none of these factors were found to act as protective factors. The findings have several implications for services for children with ASD and their families.

**Table 1:** Demographic characteristics of participants

Variable	*n* (% of sample)	Mean	SD	Range
Parent characteristics	*n* = 136			
Female	116 (85.3%)			
Male	20 (14.7%)			
Parent’s age		42.58 years	7.06 years	21–68 years
Parent’s country of origin	80.1% Ireland; 8.8% England; 5.9% Eastern European country; 2.2% Asian country, 1.5 Northern Ireland; 1.5% African country			
Education level	59% University level; 21% post leaving certificate/technical qualification; 20% secondary school level			
Employment status	35.3% carer; 33.1% full time; 23.5% part time; 6.6% other (including leave, retired, self-employed); 1.5% unemployed			
Marital status	68% married (first marriage), 10% single, 10% co-habiting with partner, 9% divorced/separated, 3% remarried			
Family annual income	34% < 30,000; 34% 31–59,000; 21% 60–89,000; 11% 90,000 and above			
Number of children		2.57	1.12	1–7
Number of children with ASD	80% 1 child with ASD; 17% 2 children with ASD; 3% have 3 children with ASD			
Child characteristics	*n* = 133			
Female	36 (27.1%)			
Male	96 (72.7%)			
Non-binary	1 (0.8%)			
Child’s age		10.45 years	3.83 years	4–18 years
ASD diagnosis	108 (81%)			
Asperger’s syndrome	21(16%)			
PDD-NOS diagnosis	4(3%)			
Co-morbid ID	42 (32%)			
Other co-morbid diagnosis e.g., ADHD	49 (37%)			
Education setting	37% in mainstream school; 27% mainstream preschool; 14% in ASD class in mainstream school; 11% special school; 5% other; 4% ASD preschool; 2% no placement; 2% home tuition			

*ID* intellectual disability, *ADHD* attention deficit hyperactivity disorder

**Table 2:** Correlations between study variables

Variables	1	2	3	4	5	6	7	8	9	10	11	12	13	14
1. Self-injurious behaviour frequency	–	.920**	.508**	.519**	.511**	− .068	− .103	− .080	− .279**	.360**	.330**	− .307**	− .137	− .156
2. Self-injurious behaviour severity		_	.445**	.530**	.389**	− .073	− .074	.075	− .256**	.352**	.312**	− .319**	− .121	− .114
3. Aggressive/destructive behaviour frequency			_	.911**	.311**	− .257**	− .235**	− .113	− .201*	.513**	.367**	− .144	− .142	− .210*
4. Aggressive destructive behaviour severity				–	.276**	− .216**	− .248**	− .125	− .201*	.508**	.380**	− .159	− .166	− .200*
5. Stereotyped behaviour frequency					_	.067	− .057	.033	− .132	.261**	.268*	− .121	− .171*	− .158
6. Positive perceptions						_	.434*	.534**	.420**	− .447**	− .403*	.321**	.390**	.499**
7. Self-efficacy							–	.449**	.365**	− .564**	− .554**	.573**	.512**	.412**
8. Adaptive coping								–	.692**	− .500**	− .557**	.616**	.614**	.502**
9. Social support									–	− .582*	− .626**	.552**	.677**	.502*
10. Parenting stress total										–	.892**	− .888*	− .604**	− .559**
11. Parent distress											–	− .653**	− .617**	− .634**
12. Family quality of life												–	.637**	.558**
13. Psychological wellbeing													–	− .548*
14. Life satisfaction														–

*p < .05; **p < .01

**Table 3:** Summary of hierarchical regression analysis of parental stress

Step	Variables	*β*	Adjusted *R*^*2*^	*F*	*ΔR *^*2*^	*F* change
1.	Income	− .163*				
	Age of child with ASD	.063				
	More than one child with ASD	.189**				
			.079	4.678*	.100	4.678**
2.	Self-injurious behaviour	− .005				
	Aggressive behaviour	.302**				
	Stereotyped behaviour	.090				
			.317	10.993**	.249	15.674**
3.	Adaptive coping	− .099				
	Social support	− .243**				
	Positive perceptions	− .098				
	Self-efficacy	− .330**				
			.649	24.892**	.327	30.123**

**p < .001, *p < .005

**Table 4:** Summary of hierarchical regression analysis of family quality of life

Step	Variables	*β*	Adjusted *R*^*2*^	*F*	*ΔR *^*2*^	*F* change
1.	Marital Status	− .028				
			− .008	.000	.000	.000
2.	Self-injurious behaviour					
	Aggressive behaviour	− .176*				
	Stereotyped behaviour	.151*				
			.069	3.379*	.098	4.505
3.	Adaptive coping	.227*				
	Social support	.380**				
	Positive perceptions	.009				
	Self-efficacy	.282**				
			.570	22.371**	.499	37.424

**p < .001, *p < .005

**Table 5:** Summary of hierarchical regression analysis of psychological wellbeing

Step	Variables	*β*	Adjusted *R*^*2*^	*F*	*ΔR *^*2*^	*F* change
1.	Income		− .121			
			− .007	.148	.001	.148
2.	Self-injurious behaviour	.054				
	Aggressive behaviour	.036				
	Stereotyped behaviour	− .163*				
			.008	1.257	.038	1.626
3.	Adaptive coping	.413**				
	Social support	.176				
	Positive perceptions	− .157*				
	Self-efficacy	.413**				
			.528	19.054*	.519	35.465**

**p < .001, *p < .005

**Table 6:** Summary of hierarchical regression analysis of life satisfaction

Step	Variables	*β*	Adjusted *R*^2^	*F*	*ΔR*^2^	*F* change
1.	Self-injurious behaviour	.042				
	Aggressive behaviour	− .019				
	Stereotyped behaviour	− .159				
			.028	2.124	.054	2.124
2.	Adaptive coping	.149				
	Social support	.224*				
	Positive perceptions	.275**				
	Self-efficacy	.126				
			.360	10.256**	.345	15.528**

**p < .001, *p < .005

**Table 7:** Coping as the moderator: moderated multiple regression analyses for adaptation outcomes

Moderator model	Parental stress	Family quality of life	Psychological wellbeing	Life satisfaction
Adaptive coping as moderator	*R* = .727; *R*^2^ = .528 *F* = 23.84**; *n* = 135	*R* = .665; *R*^2^ = .443 *F* = 25.63** *n* = 134	*R* = .649; *R*^2^ = .421 *F* = 23.42; *n* = 134	*R* = .539; *R*^2^ = .291 *F* = 15.7; *n* = 119
Variable	*P*	*P*	*P*	*P*
Family income	.011*			
Marital status		.910	.334	
More than one child with ASD	.044*			
Age of child with ASD	.026*			
Behavioural difficulties	.000**	.002**	.006*	.0134
Adaptive coping	.000**	.000**	.000**	.0000
Adaptive coping × behavioural difficulties (interaction)	.641	.196	.921	.7840

**Table 8:** Positive perceptions as the moderator: moderated multiple regression analyses for adaption outcomes

Moderator model	Parental Stress	Family Quality of Life	Psychological wellbeing	Life Satisfaction
Positive Perception as moderator	*R* = .686; *R*^*2*^ = .471 *F* = 18.52**; *n* = 132	*R* = .509; *R*^*2*^ = .259 *F* = 11.02**; *n* = 131	*R* = .389 *R*^*2*^ = .151 *F* = 5.56**; *n* = 130	*R* = .539 *R*^*2*^ = .291 *F* = 15.73 *n* = 119
Variable	*P*	*P*	*P*	*P*
Family income	.005**			
Marital status		.219	.737	
More than one child with ASD	.003**			
Older child with ASD	.836			
Behavioural difficulties	.000**	.0001**	.013*	.0134
Positive Perception	.000**	.000**	.001**	.0000
Positive perception × behavioural difficulties (interaction)	.421	.174	.349	.7840

**Table 9:** Parental self-efficacy as the moderator: moderated multiple regression analyses for adaptation outcomes

Moderator model	Parental stress	Family quality of life	Psychological wellbeing	Life satisfaction
Parental self-efficacy as moderator	*R* = .755; *R*^2^ = .569 *F* = 28.23**; *n* = 135	*R* = .575; *R*^2^ = .331 *F* = 15.92; *n* = 134	*R* = .599; *R*^2^ = .356 F = 17.94**; n = 134	*R* = .45; *R*^2^ = .203 *F* = 9.76**; *n* = 119
Variable	*P*	*P*	*P*	*P*
Family income	.0015**			
Marital status		.642	.209	
More than one child with ASD	.0012**			
Older child with ASD	.176			
Behavioural difficulties	.000**	.0005**	.070	.062
Parental self-efficacy (PSE)	.000**	.000**	.000**	.000**
PSE × behavioural difficulties (interaction)	.115	.383	.5121	.159

**Table 10:** Social support as the moderator: moderated multiple regression analyses for adaptation outcomes

Moderator model	Parental stress	Family quality of life	Psychological wellbeing	Life satisfaction
Social support as moderator	R = .744; R^2^ = .554 F = 25.86; n = 132	R = .696 R^2^ = .485 F = 29.6; n = 131	R = .569 R^2^ = .324 F = 14.95**; n = 130	R = .541 R^2^ = .293 F = 15.48**; n = 116
Variable	*P*	*P*	*P*	*P*
Family income	.047			
Marital status		.425	.951	
More than one child with ASD	.411			
Older child with ASD	.108			
Behavioural difficulties	.000**	.025	.064	.024*
Social support	.000**	.000**	.000**	.000**
Social support × behavioural difficulties (interaction)	.468	.901	.551	.333
